# The factor structure of the cardiac anxiety questionnaire, and validation in a post-MI population

**DOI:** 10.1186/s12874-022-01820-5

**Published:** 2022-12-29

**Authors:** Philip Leissner, Claes Held, Elisabet Rondung, Erik M. G. Olsson

**Affiliations:** 1 Uppsala University, Uppsala, Sweden; 2 Uppsala Clinical Research Center, Uppsala, Sweden; 3 Uppsala University Hospital, Uppsala, Sweden; 4 Mid Sweden University, Östersund, Sweden

**Keywords:** Anxiety, Cardiac, Heart, Factor analysis, Psychometrics, Validation

## Abstract

**Background:**

CVD-patients with higher levels of cardiac anxiety suffer psychologically, as well as being at increased risk for cardiac morbidity and mortality. Therefore it is important to be able to assess CA in a clinical setting. It is currently measured with the Cardiac Anxiety Questionnaire, which has conflicting findings regarding its factor structure, and it has not been validated in a Swedish population. This study aimed to examine the factor structure of CAQ and its psychometric properties in a Swedish CVD-population.

**Methods:**

Nine hundred thirty patients post-MI were recruited at different Swedish hospitals and completed the CAQ, along with several other questionnaires. Exploratory factor analysis and confirmatory factor analysis were conducted to explore factor structure and to inspect various factor solutions from previous research. Standard psychometric tests were performed for the CAQ to test its validity and reliability.

**Results:**

The exploratory analysis found a model with the factors Fear/Worry, Avoidance and Attention. The confirmatory factor analysis indicated that a 3-factor solution best fitted the data, but with certain items removed. Additionally, psychometric properties turned out acceptable in a Swedish post-MI population.

**Conclusions:**

We conclude that the original 3-factor structure of the CAQ is valid, but that the questionnaire could be revised in regard to some items. A shorter 10-items version could also be considered. We also confirm that the CAQ is a valid instrument to measure CA in a Swedish MI-population.

**Trial registration:**

The study was registered on ClinicalTrials.gov on 05/01/2012 (NCT01504191).

## Introduction

Cardiovascular disease (CVD) is the main cause of mortality world-wide [1] and suffering a CV event can be highly anxiogenic. Around 20-30% of patients experience increased anxiety after a myocardial infarction (MI) [2]. Furthermore, anxiety also serves as an independent risk factor for incident coronary heart disease and cardiac mortality [3]. Assessment of anxiety in the clinical setting is thus an important step in evaluating prognosis and offering optimal care.

Anxiety specifically triggered by fear of heart disease or heart malfunctioning is called cardiac anxiety (CA). The most common form of measuring CA in a clinical setting has been to use the Cardiac Anxiety Questionnaire (CAQ) [4]. It consists of three subscales; Fear, Avoidance and Attention. This is an instrument that has shown good psychometric properties in patients with and without CVD and in several different countries [4–10]. Many of these studies have also explored the factor structure of CAQ and proposed varying factor solutions and removal of items [5–8, 10]. For example, previous work has suggested adding Reassurance Seeking as a fourth subscale [5–7], combining the factors Fear and Attention in one subscale and removing 4 items [10], and removing 8 items [8]. The studies also differ in varying degree to the use of methodologies (See Table 1 for an overview). While it seems uncertain what the exact dimensions of CAQ are, those of the original are still being used as standard choice around the world.

**Table 1:** Overview of previous studies

Study	Language	***n***	EFA^a^	CFA	Split	Factors
Eifert et al. (2000) [4]	English	188	PCA	No	n/a	3
Marker et al. (2008) [5]	English	658	Common	Yes	No	4
Dragioti et al. (2011) [8]	Greek	598	PAF	Yes	Yes	3
Van Beek et al. (2012) [6]	Dutch	237	Yes	Yes	No	4
Fischer et al. (2012) [9]	German	2396	No	Yes	n/a	3
Sardinha et al. (2013) [10]	Portuguese	98	PCA	No	n/a	2
Israel et al. (2017) [7]	English	229	No	Yes	n/a	4

^a^*PCA* Principal Component Analysis, *Common* Common Factor Analysis, *PAF* Principal Axis Factoring, *Yes* EFA conducted but method not specified

The Swedish version of CAQ has not yet been validated and it remains unclear whether it shares the psychometric properties of the original. Thus, there were two main aims with the current study. First, to perform an exploratory factor analysis and to compare this suggestion along with all previously suggested factor solutions in a confirmatory analysis. Second, to test the validity and reliability of the Swedish translation of the CAQ.

## Methods

### Study design and participants

This study was part of the U-CARE Heart trial, a multi-centre randomised control trial that evaluated the effect of internet-based cognitive behavioural therapy in patients with a recent MI. The study design, procedure, intervention, and results have been reported elsewhere [11, 12].

Nine hundred thirty-five participants with MI were screened. Out of these, 930 participants completed CAQ. Patients who reported a score of > 7 on one or both subscales of the Hospital Anxiety and Depression Scale (HADS) [13] were randomized to either internet-based CBT (*n* = 117) or a control group receiving usual care (*n* = 122).

### Procedure

Eligible participants (< 75 years old, MI within 3 months) were recruited during routine visits 1-8 weeks following their MI in 25 Swedish cardiac clinics from September 2013 to December 2016. The questionnaires were answered online 8-10 weeks after discharge. Those who were participating in the clinical RCT answered questionnaires at several time points.

### Measures

#### Sociodemographic characteristics and cardiac risk factors

Self-reported data of educational level, country of birth, physical activity and smoking was collected in a customized questionnaire. Data on length, weight, hypertension, diabetes and previous MI were collected from the SWEDEHEART register. Obesity data was self-reported as length and weight. BMI was calculated (kg/m^2^) and *Obesity* was defined as a BMI equal to or higher than 30.

#### Cardiac anxiety questionnaire

CAQ measures CA [4]. It comprises 18 items and can be divided in three subscales (Fear, 8 items; Avoidance, 5 items; Attention, 5 items). Each item is rated on a 5-point Likert scale with scores ranging from 0 (never) to 4 (always). A high score indicates a greater number of symptoms, greater frequency, or both. It is suggested [4] to divide the total score by the number of items, making the score range from 0 to 4. The Swedish version used in this study was translated by the authors of the RCT, through translation and back-translation.

#### Hospital anxiety and depression scale (HADS)

HADS assesses symptoms of anxiety and depression in a clinical setting [13]. It consists of 14 items, where 7 measure symptoms of anxiety and 7 measure symptoms of depression. Each item is rated on a 4-point Likert scale ranging from 0 to 3, total score range 0-42. A high score indicates a greater number of symptoms, greater intensity, or both. It has reported good psychometric properties and is a common choice for measuring anxiety and depression.

#### Behavioral activation for depression scale, short form (BADS-SF)

BADS measures escape and avoidance behaviour in depression. The Short Form consists of nine items that can be divided in two subscales (Avoidance, 3 items; Activation, 6 items). Each item is rated on a 7-point Likert scale, ranging from 0 (not at all) to 6 (completely), total score range 0-54. A high score indicates a low presence of escape and avoidance behaviours. Both versions have reported good reliability and validity [14, 15].

#### Post traumatic checklist, civilian version (PCL-C)

The civilian version of PCL has been confirmed as of having good psychometric properties [16]. It consists of three subscales (Re-experiencing, 5 items; Avoidance or numbing, 7 items; Arousal, 5 items) and the 17 items are rated on a 5-point Likert scale from 1 (not at all) to 5 (extremely), total score range 17-85. A high score indicates a greater number of symptoms, greater intensity, or both.

#### Montgomery Åsberg depression rating scale, self-rating version (MADRS-S)

MADRS measures the most commonly occurring symptoms of depression [17]. It consists of 9 items, rated on a Likert scale ranging from 0 to 6, total score range 0-54. A high score indicates a greater number of symptoms, greater intensity, or both. The self-administered version has good psychometric properties [18].

### Statistical analyses

Stata Software Package (version 17.0) was used to perform all statistical analyses. Out of the 930 individuals who completed the CAQ, there were missing values (1-3) for PCL, BADS and MADRS-S. If a participant had a missing value for a questionnaire or a subscale, they were excluded from any analysis including that questionnaire or subscale.

As the aim if this study was to conduct both an exploratory factor analysis (EFA) and a confirmatory factor analysis (CFA), the sample was split in two random equal halves [19]. The EFA was conducted on the first half and the CFA on the second. These halves will be referred to as the exploratory split (ES) and confirmatory split (CS). All tests of validity and reliability were performed on the CS.

#### Factor analysis

Sampling adequacy was measured with the Keyser-Meyer-Olkin (KMO) test, and was used to determine if the correlation matrix was factorable. Due to the ordinal level of the data, a polychoric correlation matrix was calculated for the basis of the EFA [20]. Common factor analysis was conducted, as the aim of this study was to observe underlying latent variables [21]. Suitable proportion of factors to retain was judged by the Kaiser criterion (Eigenvalue > 1), visual inspection of Scree Plot [22],Velicer’s map criteria [23] and Parallel Analysis [24]. Interpretability and adherence to theory was also considered in this process. Due to the internal correlation of the factor structure, oblique (promax) rotation was used. Saliency of item loadings on factors were determined for significant coefficients ≥.24, calculated based on current sample size [25]. Items were sequentially removed if they had no salient factor loading or if they loaded across more than one factor, and the analysis was re-run. A factor was considered adequate when it consisted of at least 3 items with salient loadings, a Cronbach’s alpha of ≥.70 and deemed theoretically meaningful [26].

To test model fit, a Confirmatory Factor Analysis (CFA) was conducted using mean-and-variance corrected statistics for Structural Equational Modelling. The model fit was assessed based on the values of root mean square error of approximation (RMSEA), comparative fit index (CFI), and Tucker–Lewis index (TLI). To conclude a good model fit, the value of RMSEA should be close to <.06 and CFI and TLI should be close to >.95 [27]. CFA was conducted for all factor solutions represented in previous literature, the model generated by the EFA as well as a 1-factor solution.

#### Validity and reliability

All tests of reliability and validity were performed on the new factor solution generated by the EFA and the original model by Eifert. The value of Cronbach’s alpha was used in the ES to determine internal consistency of the full CAQ, as well as the individual subscales. Using the Spearman correlation coefficient, convergent and discriminant validity was examined for the full questionnaire and the CAQ subscales against HADS, MADRS-S, BADS and PCL-C, as well as their individual subscales. Reliability of the questionnaire was further examined by test-retest, using the Spearman correlation coefficient (control group of RCT, 5 weeks between measurement points). All correlations were made using both the ES and the CS.

## Results

### Study population

A total of 930 participants (711 males) participated in the data collection; mean age being 62.2 years (SD = 8.1, min = 31, max = 75). There was no difference in age or in any other background or clinical characteristic between the ES (*n* = 465) and CS (*n* = 465) (all ps = > 0.17). See Tables 2 and 3.

**Table 2:** Characteristics of subjects (*N* = 930)

Variables	*Total % (n)*	*Exploratory Split (n = 465)*	*Confirmatory Split (n = 465)*
Cardiovascular risk factors			
Physical inactivity^a^	75% (705)	74% (347)	77% (358)
Obesity^f^	23% (211)	23% (121)	23% (108)
Smoking^b^	23% (214)	25% (118)	21% (100)
Diabetes^d^	15% (142)	17% (78)	14% (64)
Hypertension^e^	42% (387)	43% (199)	41% (191)
Previous myocardial infarction^d^	11% (103)	12% (55)	10% (49)
Sociodemographic factors			
Women	24% (219)	24% (112)	23% (108)
Education			
Elementary school	20% (188)	21% (97)	20% (93)
High School	37% (348)	38% (180)	36% (169)
< 3y University	20% (183)	20% (94)	19% (91)
> 3y University	23% (211)	21% (97)	24% (114)
Born in Sweden	91% (842)	92% (429)	89% (417)

^a^1 missing data points

^b^2 missing data point

^c^3 missing data points

^d^31 missing data points

^e^33 missing data points

^f^49 missing data points

**Table 3:** Characteristics of subjects (*N* = 930)

Variables	*Total Mean (SD)*	*Exploratory Split*	*Confirmatory Split*
CAQ – Total	0.9 (0.6)^a^	1.0 (0.6)	0.9 (0.6)
CAQ – Fear	0.9 (0.8)^a^	1.0 (0.8)	0.9 (0.8)
CAQ – Avoidance	1.0 (0.8)^a^	1.0 (0.8)	1.0 (0.8)
CAQ – Attention	0.8 (0.6)^a^	0.9 (0.6)	0.8 (0.6)
HADS – Anxiety	5.0 (4.2)	5.1 (4.3)	5.0 (4.1)
HADS – Depression	3.9 (3.7)	4.0 (3.9)	3.8 (3.6)
MADRS-S^d^	7.1 (7.9)	7.3 (8.2)	6.9 (7.6)
BADS – Total^b^	38.5 (10.7)	38.2 (10.7)	38.8 (10.4)
BADS – Avoidance	15.5 (3.5)	15.4 (3.5)	15.6 (3.3)
BADS – Activation^b^	23.0 (8.5)	22.8 (8.6)	23.2 (8.4)
PCL – Total^d^	25.0 (10.1)	25.2 (10.6)	25.7 (9.6)
PCL – Re-experiencing^b^	6.8 (2.8)	6.9 (2.9)	6.7 (2.7)
PCL – Avoidance or numbing^d^	10.0 (4.3)	10.1 (4.4)	10.0 (4.2)
PCL – Arousal^c^	8.2 (4.0)	8.3 (4.2)	8.0 (3.8)

Questionnaires: *HADS* Hospital Anxiety and Depression Scale, *CAQ* Cardiac Anxiety Questionnaire, *PCL* Post-traumatic Checklist, Civilian version, *BADS-SF* Behavioral Activation for Depression Scale, Short Form, *MADRS-S* Montgomery Åsberg Depression Rating Scale – Self-administered

^a^Scores on CAQ are mean values instead of total values

^b^1 missing data points

^c^2 missing data point

^d^3 missing data points

### Factor structure

#### Exploratory factor analysis

**Fig. 1:**
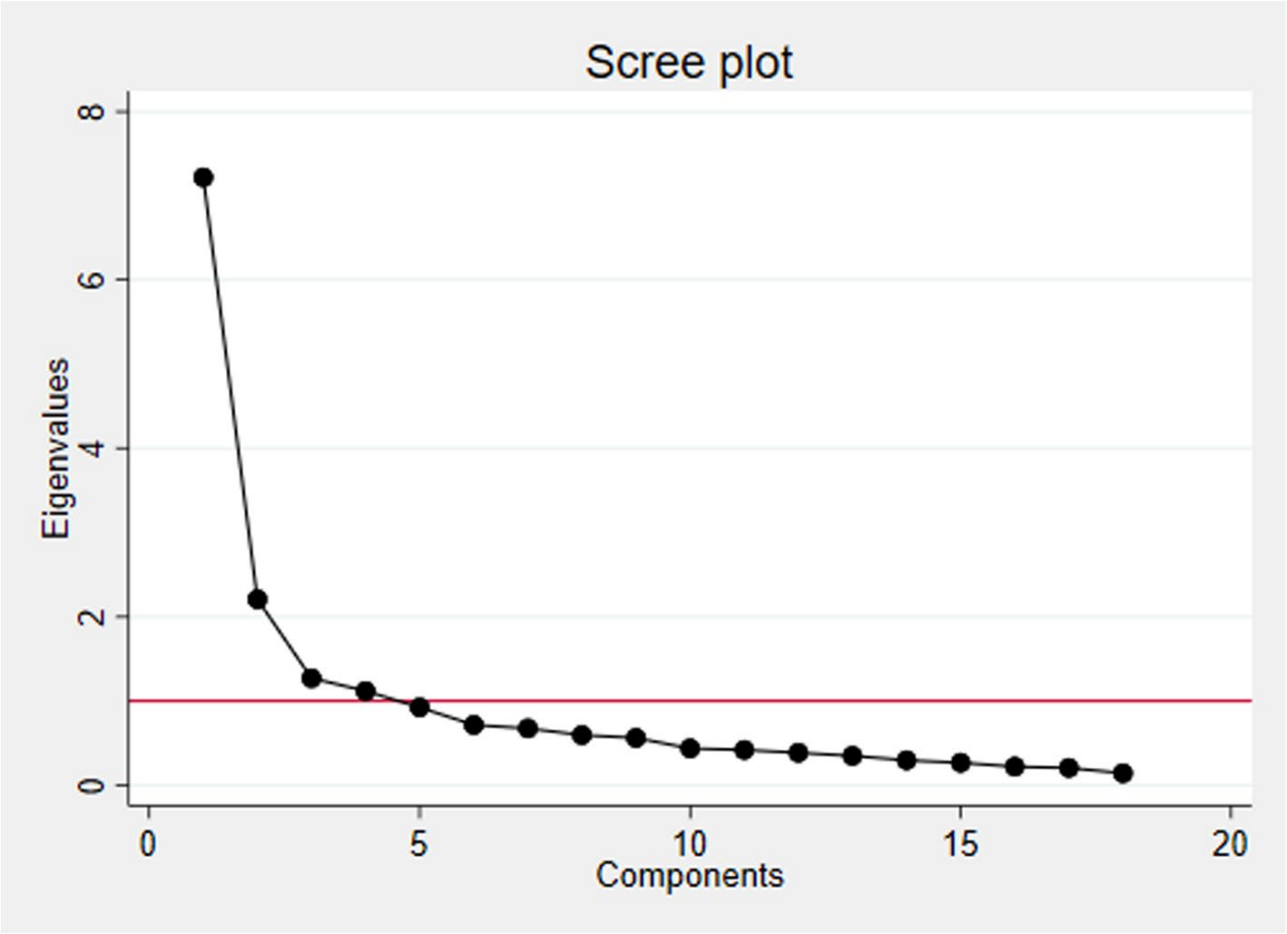
Scree plot of eigenvalues after EFA. Horizontal line is set at eigenvalue = 1

**Fig. 2:**
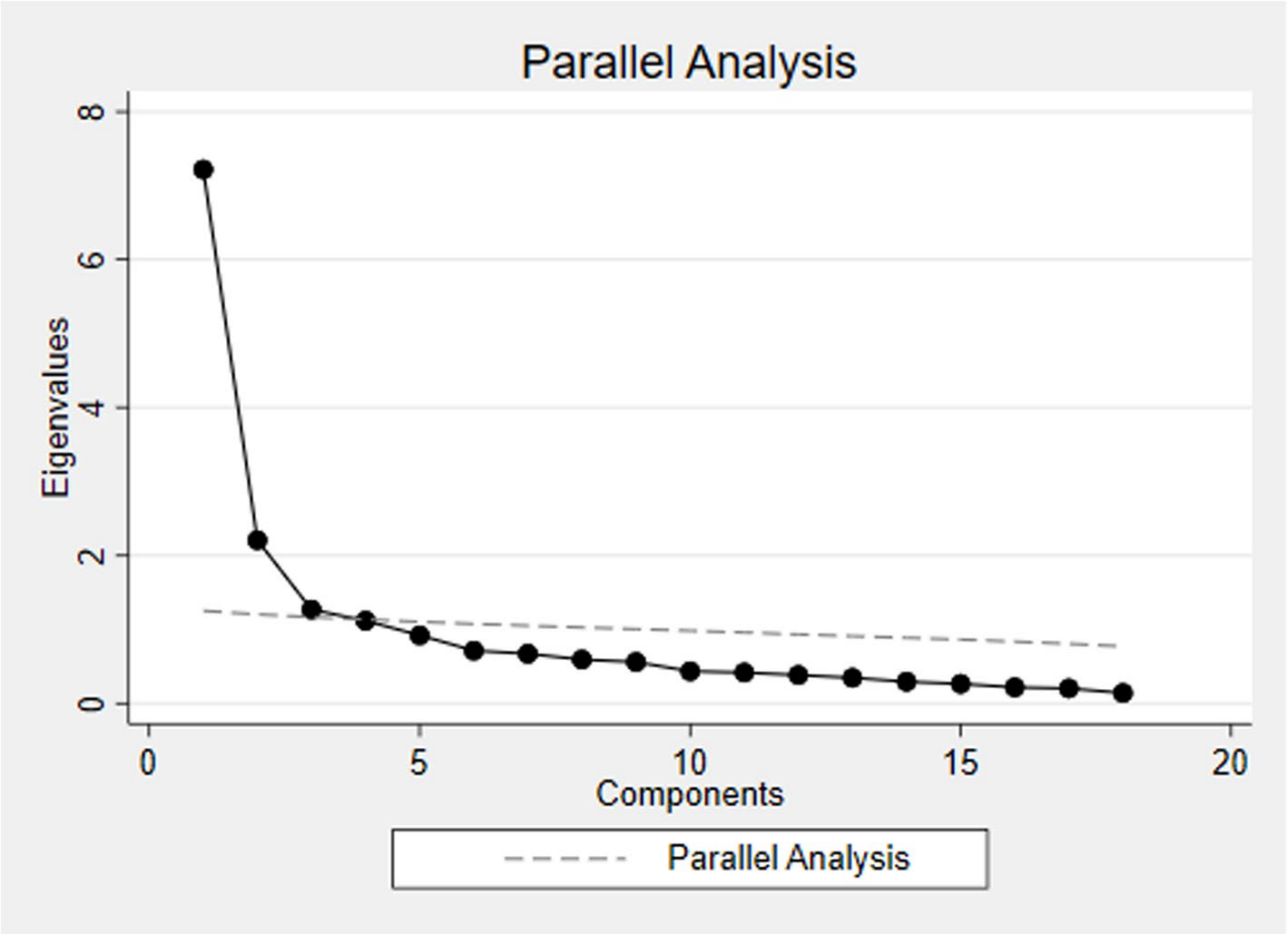
Parallel Analysis of EFA

The KMO value of 0.88 verified the sampling adequacy and indicated that the correlation matrix was adequate for conducting factor analysis [28]. Two factors were identified with eigenvalue > 1. Breaks in the scree plot can be seen at the third and sixth factor (see Fig. 1). Velicer’s map criteria suggested retaining two factors and the parallel analysis determined the upper limit of factors to rotate and evaluate at 8 (see Fig. 2). As such, factor solutions containing 2-8 factors were rotated and compared. Considering interpretability, theoretical congruence, internal consistency, a minimum of three items per factor and amount of information retained, the 3-factor solution was deemed most reasonable. This model explained a cumulative variance of 66% (See Table 4).

**Table 4:** Common factor analysis

Item	Fear/Worry	Avoidance	Attention
*Fear*			
11. I feel safe being around a hospital, physician, or other medical facility^a^	**0.56**	0.18	0.10
14. When I have chest discomfort or I feel my heart is beating fast I worry that I may have a heart attack^a^	**0.84**	0.01	0.09
15. When I have chest discomfort or I feel my heart is beating fast I have difficulty concentrating on anything else^a^	**0.76**	0.07	0.21
16. When I have chest discomfort or I feel my heart is beating fast I get frightened^a^	**0.88**	−0.02	0.10
17. When I have chest discomfort or I feel my heart is beating fast I like to be checked out by a doctor^a^	**0.93**	−0.10	−0.09
18. When I have chest discomfort or I feel my heart is beating fast I tell my family or friends^a^	**0.61**	−0.02	−0.16
*Avoidance*			
2. I avoid physical exertion^b^	0.01	**0.92**	−0.07
5. I take it easy as much as possible^b^	0.21	**0.53**	−0.03
7. I avoid exercise or other physical work^b^	−0.13	**0.97**	0.01
9. I avoid activities that make my heart beat faster^b^	0.09	**0.82**	0.08
12. I avoid activities that make me sweat^b^	0.00	**0.91**	−0.01
*Attention*			
1. I pay attention to my heart beat^c^	0.10	0.01	**0.76**
3. My racing heart wakes me up at night^c^	0.22	0.05	**0.56**
6. I check my pulse^c^	0.04	−0.03	**0.54**
8. I can feel my heart in my chest^c^	0.14	−0.03	**0.70**
*Removed*			
4. Chest pain/discomfort wakes me up at night^c^			
10. If tests come out normal, I still worry about my heart^a^			
13. I worry that doctors do not believe my chest pain/discomfort is real^a^			

Rotated (promax) EFA. A loading > .24 is displayed in bold

^a^Part of the factor Fear in the original model

^b^Part of the factor Avoidance in the original model

^c^Part of the factor Attention in the original model

Four items (4, 10, 13 and 18) had salient cross-loadings and were removed sequentially, starting with the strongest cross-loading. All of the cross-loadings were between Factor 1 and Factor 2. As three of the items had been removed (4, 10 and 13) the fourth item (18) was no longer cross-loaded. Two of the removed items (10 and 13) originated from the factor Fear and one from the factor Attention (4) in Eifert’s original model [4]. Factor 1 consisted of the items 10, 14, 15, 16, 17 & 18 originally from the factor Fear and was named Fear/Worry. Factor 2, Avoidance, was identical to the same factor in all previous models (containing items 2, 5, 7, 9 and 12). Factor 3, Attention, contained four items (1, 3, 6 & 8) from the original model.

#### Confirmatory factor analysis

As presented in Table 5, The CFA showed that only the model suggested by Dragioti et al. and the model generated from the EFA had close to an acceptable fit to the data. Worst model fit statistics were demonstrated by the 1-factor solution. The various 4-factor solutions were slightly superior to the original model by Eifert et al. [4]. The 2-factor solution by Sardinha et al. [10] also demonstrated a poor fit to the data.

**Table 5:** Confirmatory factor analysis

Model	Model specification	χ^2^ (*df)*	RMSEA	CFI	TLI
1-factor	1 factor, 18 items	1631.197^***^ (*135*)	.154	.664	.619
Eifert, 2000	3 factors, 18 items	586.14^***^ (*132*)	.086	.898	.882
Marker, 2008	4 factors, 18 items	546.17^***^ (*129*)	.083	.906	.889
Dragioti, 2011	3 factors, 10 items	110.72^***^ (*32*)	.073	.970	.958
Van Beek, 2012	4 factors, 18 items	559.01^***^ (*129*)	.085	.903	.886
Sardinha, 2013	2 factors, 14 items	409.22^***^ (*76*)	.097	.911	.893
Israel, 2017	4 factors, 18 items	549.09^***^ (*129*)	.084	.906	.888
Generated by EFA	3 factors, 15 items	317.05^***^ (*87*)	.075	.938	.925

Table includes: Root mean squared error of approximation (RMSEA), Comparative fit index (CFI), Tucker-Lewis Index (TLI) & Chi2 with n = degrees of freedom (χ^2^ (*df*))

^***^P < 0.001

### Internal consistency

Internal consistency for all the items in CAQ was ɑ = 0.89 and for Eifert’s 3 subscales it was for Fear ɑ = .89; for Attention ɑ = .70 and for Avoidance ɑ = .87. Internal consistency for the full scale in the solution generated by the EFA was ɑ = 0.89 and for the subscales it was Fear/Worry ɑ = .86; Avoidance ɑ = .87; Attention ɑ = .67.

### Test-retest reliability

The correlation between baseline and a retest 5 weeks later was *ρ* = .75 for the CAQ total score. For Eifert’s three subscales, Fear, Avoidance and Attention the correlations were *ρ* = .64, .76 and .75 respectively. The total score for the 15 item solution generated by the EFA had a test-retest correlation of *ρ* = .75, while its subscales Fear/Worry, Avoidance and Attention had a test-retest correlation of *ρ* = .63, .74, .75, respectively.

### Convergent & discriminant validity

The total scores of the original CAQ model and the CAQ model proposed by the EFA correlated very strongly (*ρ* = .99). Subsequent descriptions of correlations in this section will refer only to the CAQ model generated by the EFA. The CAQ total score correlated significantly with other anxiety questionnaires (HADS Anxiety, *ρ* = .66; PCL-C, *ρ* = .70) and also significantly but to a lesser degree with depressive questionnaires (HADS Depression, *ρ* = .60; BADS, *ρ* = −.56; MADRS-S, *ρ* = .63). Within the CAQ, the highest correlations were found between the CAQ total score and Fear/Worry (*ρ* = .89) and the CAQ total score and Avoidance (*ρ* = .76). Avoidance showed a weaker correlation with the other two avoidance-scales (BADS - Avoidance *ρ* = −.35 and PCL - Avoidance or numbing *ρ* = .47). Fear/Worry was the factor that had the highest correlations with other anxiety measures (HADS-A *ρ* = .61 and PCL *ρ* = .62). The subscale Attention demonstrated weaker correlations with all other measures (*ρ* ≤ .52). (See Table 6 for overview of correlations between questionnaires and subscales).

**Table 6:** Correlation of questionnaires and subscales

Questionnaire	CAQ Tot (Eifert)	CAQ Tot (EFA)	CAQ F/W (EFA)	CAQ Avoid (EFA)	CAQ Att (EFA)
CAQ – Total (Eifert)	1				
CAQ – Total	0.99	1			
CAQ – Fear/Worry	0.89	0.89	1		
CAQ – Avoidance	0.73	0.76	0.46	1	
CAQ – Attention	0.74	0.73	0.56	0.37	1
HADS – Anxiety	0.66	0.63	0.61	0.37	0.51
HADS – Depression	0.60	0.57	0.51	0.41	0.44
HADS – Total	0.67	0.64	0.60	0.42	0.50
MADRS-S	0.63	0.60	0.56	0.41	0.44
PCL – Re-experiencing	0.64	0.62	0.59	0.37	0.52
PCL – Avoidance or Numbing	0.65	0.62	0.55	0.47	0.45
PCL – Arousal	0.60	0.57	0.53	0.39	0.44
PCL – Total	0.70	0.67	0.62	0.46	0.52
BADS – Activation	−0.46	− 0.45	− 0.40	− 0.45	− 0.31
BADS – Avoidance	− 0.59	− 0.57	− 0.55	− 0.35	−0.42
BADS – Total	−0.56	−0.54	− 0.45	−0.47	− 0.37

All correlations are significant on the level of *p* < .001

*F/W* Fear/Worry, *Avoid* Avoidance, *Att* Attention

## Discussion

The EFA resulted in a new model with only minor alterations in regard to the original factor structure of CAQ [4], in the removal of three items. The original model showed questionable fit in the CFA, while the models with reduced amount of items showed a better fit to the data. Adding Reassurance Seeking as a fourth factor did not improve the fit of the model, nor was this structure supported by exploratory analysis. Additionally, the CAQ showed acceptable psychometric properties in a Swedish population of post-MI patients.

### Psychometric validation

The full questionnaire exhibited excellent internal consistency suggesting that the Swedish translation of CAQ still measures a singular coherent structure. The new subscales also demonstrated a good internal consistency, except for Attention which was just below the desired cut-off. Test-retest reliability was good for both the full scale as well as the individual subscales which also indicates that the questionnaire is stable over time. The convergent correlations with PCL-C and HADS-anxiety, and the fact that the correlations with depressive indexes were lower, suggest that the CAQ still measures symptoms of anxiety. These findings point to that the CAQ works psychometrically well in a Swedish post-MI population.

### Exploratory factor analysis

In the EFA, four items (items 4, 10, 13 and 18) were initially cross-loaded between the factors Fear/Worry and Attention. Three of them were removed and one lost its cross-loading (item 18) during this process, and was thus retained in the model.

Item 4 was originally part of the factor Attention. However this item rather seem to tap into sleep disturbance than that of hypervigilance or monitoring. This could be a reflection of the common occurrence of sleep disturbance within anxiety disorders [29]. However, it could also be that this item represents some other aspect of anxiety. It also describes a sudden onset of chest pain/discomfort. A sudden onset of discomfort is also a common symptom of Panic Disorder, which an early study suggested CA to be a variation of [30]. Possibly this item describes a shared trait with Panic Disorder. Furthermore, this items has been found in varying factors or been deleted in previous analyses [5, 8, 10]. Another item that demonstrates a similar quality (item 3) is included in the model generated in the EFA. When rotating the 4-factor solution these two items loaded on a fourth factor, but as only two items in a factor is unacceptable [26] this model was discarded. As such, it is unclear whether these two items are part of the Attention aspect of CA or if they describe something else. It would be interesting for future studies to investigate the role of sleep disturbance and symptoms of panic in relation to CA.

Item 10 describes a tendency to worry, even when evidence against the need for worry has been demonstrated. This item demonstrated the strongest cross-loading in the EFA and was removed first. This suggests that its content relates both to Fear/Worry and to Attention. Both worrying and focused attention are cognitive processes, and while fear and worry are closely related concepts, so is hypervigilance and worry. As some of the items in the factor Fear/Worry describes a more emotional aspect of anxiety, rather than cognitive, this could be an explanation of the shared relation with the more cognitive factor Attention. However, in all previous studies of EFA on the CAQ this item has had a single salient loading on the factor describing Fear/Worry [4–8, 10]. It could be further theorised whether the questionnaire would benefit from a clearer distinction between cognitive and emotional aspects.

Item 13 concerns both worry and chest pain. However, the worry does not focus on whether the chest pain is dangerous or not, but rather concerns if others could be trusted. As such, it should be reconsidered whether this is a mark of CA or something else, and if it should be permanently removed from the questionnaire.

It is worth considering that item 10 and 13 were both removed in this study and in the study by Dragioti et al. [8]. This could be an indication that these items suffer from issues with translation. However, this idea is not supported by the study by Sardinha et al. [10], where neither of the four removed items were 10 or 13.

The factor Fear/Worry was reduced by two items from the original model. However, it is still the largest subscale and this modification should only have minor implications. This notion is supported by the correlation with the total measure of CAQ, suggesting that it continues to be a valid and central part of the concept CA. Fear/Worry also correlated more strongly with other measures of anxiety, which could indicate that it is also closer to the general concept of anxiety than the other factors.

The factor Avoidance has included the same items in every study of CAQ to date, making it the most robust of all factors. Still, it could be criticised for its lack of specificity. The items don’t specify the reason for avoidance of physical activity. An individual who avoids exercise may get high scores even if they do not avoid it for anxiety-related reasons.

Similar to Fear/Worry, the factor Attention also has fewer items but remains otherwise unchanged. Whether or not waking up at night is a part of attention, the remaining items seem to be a valid part of CAQ. Still, much like the factor Avoidance, these items could benefit from being more specific in regard to what drives the increased attention. Additionally, this factor demonstrated some problems with internal consistency being below the desired cut-off [26]. This is possibly the result of it being the smallest factor, as fewer items makes internal consistency go down. However, in this case, the advantages of adhering to theory and not underfactoring outweighs the disadvantages of a slightly lower internal consistency (.67 < .70).

### Confirmatory factor analysis

In summary, the model with best fit was the 10-item version by Dragioti et al. [8], followed by the 3-factor solution generated in the EFA. The models that showed inferior fit to the data were the 1-factor solution, the three different 4-factor solutions [5–7], the reduced model by Sardinha et al. [10] and – interestingly – the original model by Eifert et al. [4].

The 3-factor solution by Dragioti et al. has eight removed items and its superior fit to the data could be a reasonable suggestion for a short-form of the CAQ. The superior fit of the 3-factor solution over that of the original version suggests that the validity of the questionnaire could benefit from a removal or change of some of the items. The three four-factor solutions did not demonstrate an adequate fit to the data, adding doubt to the suggestion of the addition of Reassurance Seeking to the questionnaire, although it is a theoretically sound suggestion.

### Strengths and limitations

A limitation is that the study population included patients with a recent MI. As such, the factor solution generated in the EFA may not be generalizable to other populations. However, in regard to the psychometric properties, the instrument has been found valid and reliable in groups with non-cardiac chest pain as well [5, 7–9].

As demonstrated in Table 1, the methodologies of previous studies are varying. Some of them use principal components analysis or principal axis factoring to extract the factors while others perform both EFA and CFA in the same sample. Additionally, they rarely report if the analyses have been modelled for ordinal data. As such, this is the first study of the CAQ that has combined both EFA and CFA and followed proper statistical considerations.

## Conclusion

This study has contributed with a thorough statistical analysis and overview of the factor structure of the CAQ. The original 3-factor structure proposed by Eifert et al. [4] has been proven reasonable but could benefit from modifications. The previously proposed 10-item solution by Dragioti et al. [8] could also be considered as a valid short form for the CAQ. Additionally, it is concluded that the CAQ shows acceptable psychometric properties in a Swedish population of post-MI patients.

## Data Availability

The datasets generated and/or analysed during the current study are not publicly available due to the General Data Protection Regulation (2016/679) protecting privacy but are available from the corresponding author in an aggregated format on reasonable request.

## Abbreviations

BADS: Behavioral Activation for Depression Scale
CA: Cardiac Anxiety
CAQ: Cardiac Anxiety Questionnaire
CFA: Confirmatory Factor Analysis
CFI: Comparative Fit Index
CS: Confirmatory Split
CVD: Cardiovascular Disease
EFA: Exploratory Factor Analysis
ES: Exploratory Split
HADS: Hospital Anxiety and Depression Scale
MI: Myocardial Infarction
KMO: Keiser-Meyer-Olkin
MADRS: Montgomery Åsberg Depression Rating Scale
PCL-C: Post Traumatic Checklist, Civilian version
RMSEA: Root mean square error of approximation
TLI: Tucker-Lewis Index
